# Supplementary material for: Integrating In Vitro Biopharmaceutics into Physiologically Based Biopharmaceutic Model (PBBM) to Predict Food Effect of BCS IV Zwitterionic Drug (GSK3640254)
Source: Pharmaceutics. 2023 Feb 3;15(2):521. doi: 10.3390/pharmaceutics15020521 (PMC9965536; doi:10.3390/pharmaceutics15020521)
Supplement: Supplementary file 1 [file pharmaceutics-15-00521-s001.zip › pharmaceutics-2185216-supplementary.pdf]

Supplementary Materials

Integrating In Vitro Biopharmaceutics into Physiologically Based Biopharmaceutic Model (PBBM) to Predict Food Effect of BCS IV Zwitterionic Drug (GSK3640254)

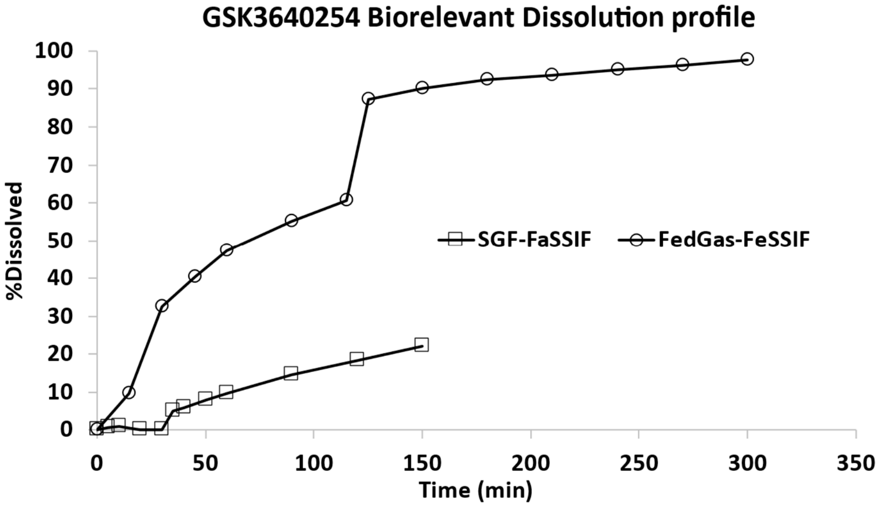

Figure S1. Dissolution profile of GSK3640254 IR tablet (150 mg) in biorelevant media. Experimental details: USP 2, 50 rpm, Fasted state: Media: 250mL SGF up to 30mins, then 250mL FaSSIF; Fed state: Media: 250mL FedGAS (pH3) up to 120mins, then 250mL FeSSIF added.

Total concentration

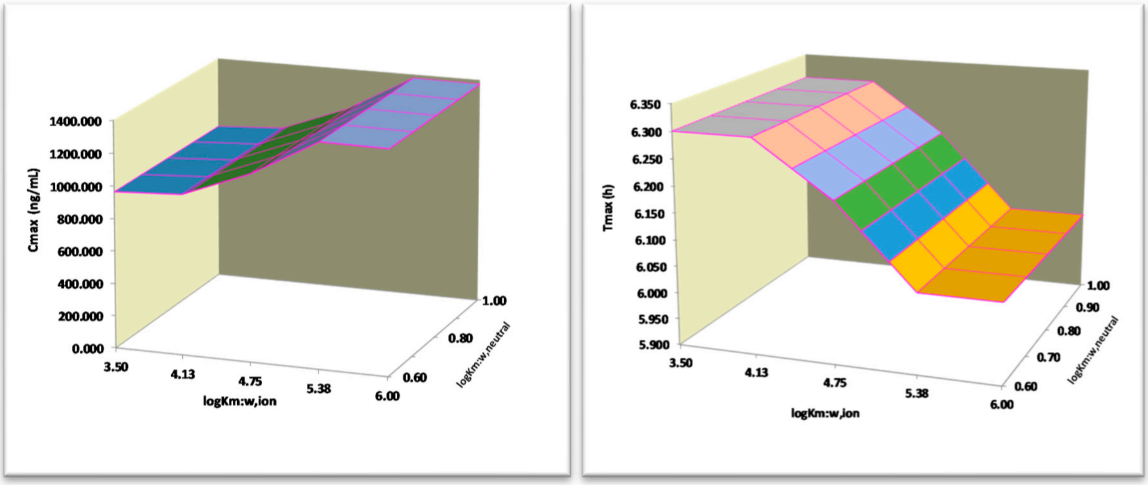

Free fraction

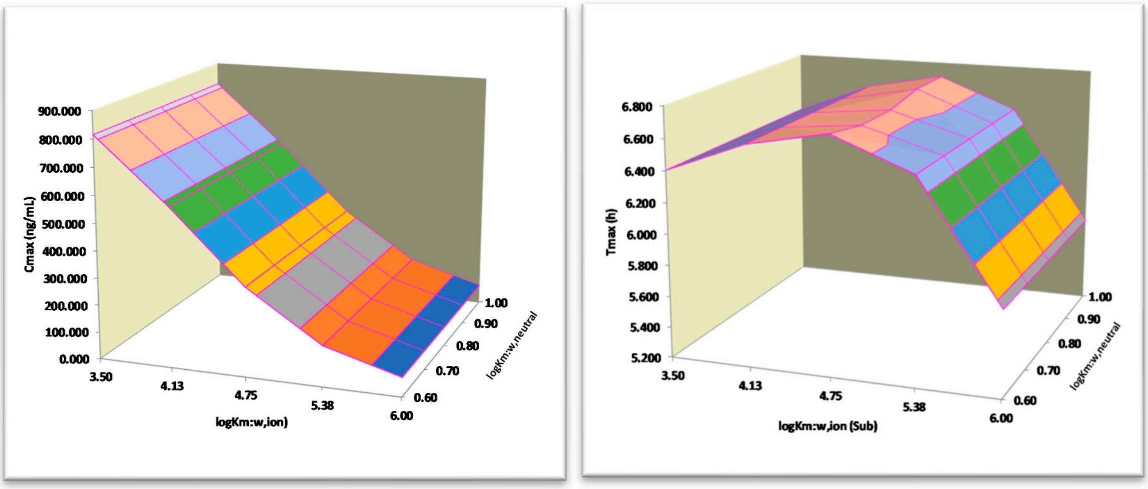

Figure S2. Parameter Sensitivity Analysis (PSA) results for LogKM:W using the total concentration and the free fraction using Ptrans,0 predicted from the in-built function in Simcyp® v.20.

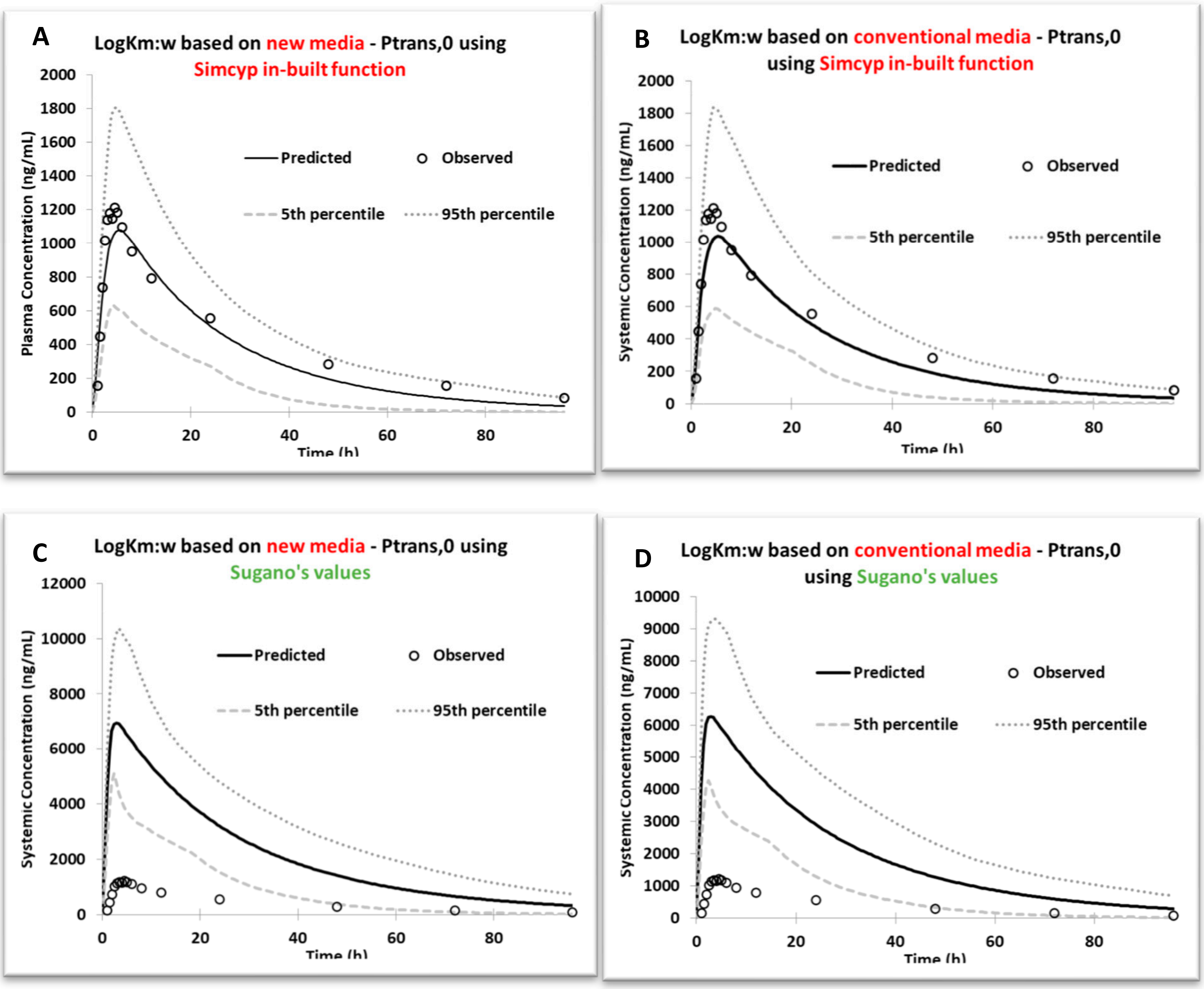

Figure S3. Impact of LogKm:w values derived from different set of biorelevant media and the LogP-to-$P_{trans,0}$ correlation function used to predict $P_{trans,0}$, on the prediction performance of the model. The predicted profiles plotted against the observed plasma concentration time profile of GKS254 following administration of single dose 200 mg IR Tablet under fed state. A) LogKm:w values used were estimated using the new media and the default in-built correlation function in Simcyp®; B) LogKm:w values used were estimated using the convetional media and the default in-built correlation function in Simcyp®; C) LogKm:w values used were estimated using the new media and Sugano's [1] values used in correlation function in Simcyp®; D) LogKm:w values used were estimated using the new media and Sugano's values used in correlation function in Simcyp®; New media refers to FaSSIF and FeSSIF media containing also oleic acid and cholesterol whereas conventional media refers to the typical FaSSIF/FeSSIF media containing Taurocholate and phosphatidylcholine.

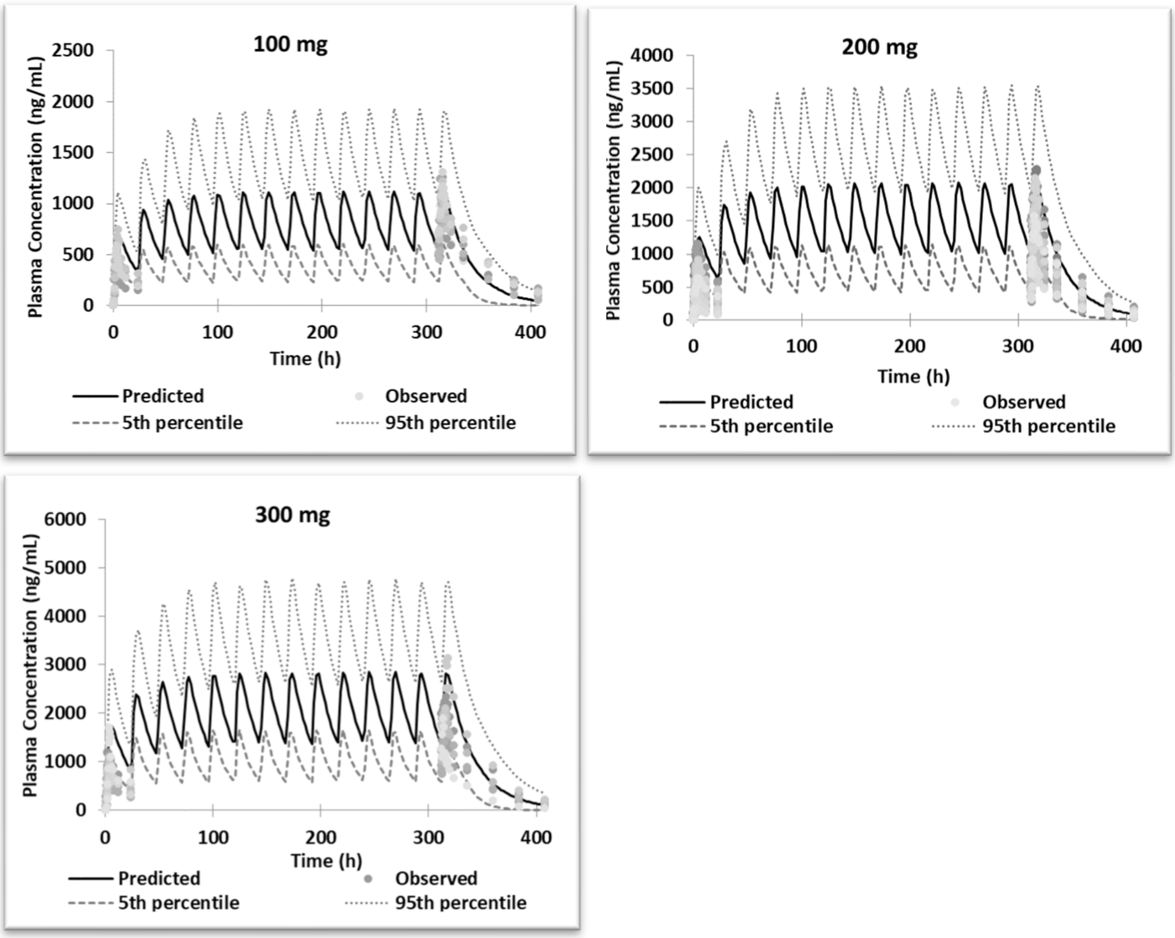

DAY14	100mg			200mg			320mg		
	Obs.	Pred.	Obs./Pred.	Obs.	Pred.	Obs./Pred.	Obs.	Pred.	Obs./Pred.
Cmax (μg/mL)	1.18	1.06	1.11	1.4	2	0.7	2.16	2.71	0.8
AUC(μg/mL h)	17.5	29.8	0.59	21.5	55.4	0.39	32.0	76.3	0.42

Figure S4. Performance verification of the PBBM model against FTIH data [2] using $P_{trans,0}$ value (5562) optimized based on ADME single dose study [3]. The predicted profiles plotted against the observed plasma concentration time profile of GKS254 following administration of once daily dose (100 – 320 mg) IR Tablet with moderate-fat meal in healthy volunteers.

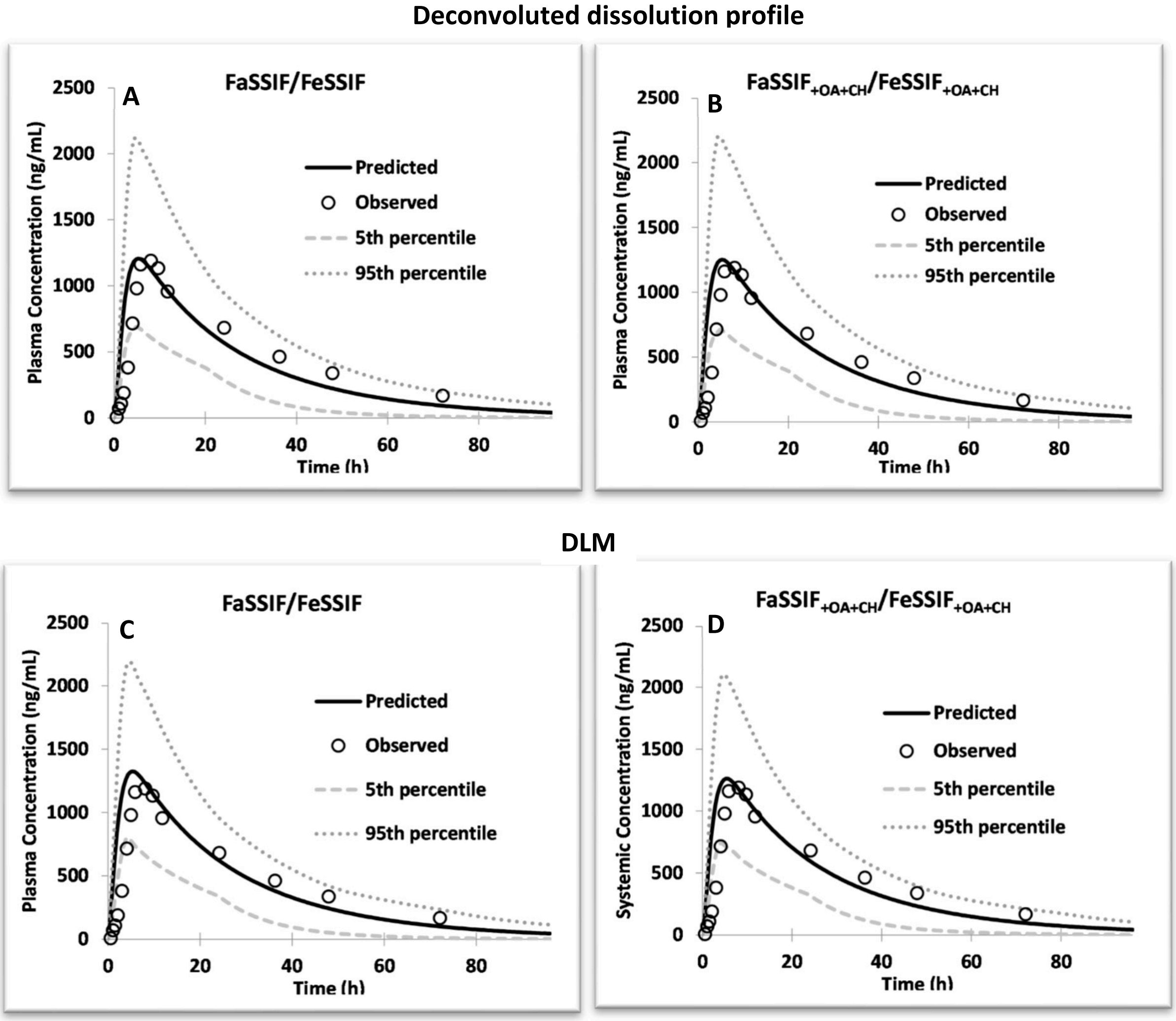

Figure S5. Comparison of model predictions against clinical data [3] using deconvoluted dissolution profile (A: $LogK_{M:W}$ estimated from FaSSIF/FeSSIF media; B: $LogK_{M:W}$ estimated from $FaSSIF_{+OA+CH}$/$FeSSIF_{+OA+CH}$ media) and Dissolution Layer Model (DLM) (C: $LogK_{M:W}$ estimated from FaSSIF/FeSSIF media; D: $LogK_{M:W}$ estimated from $FaSSIF_{+OA+CH}$/$FeSSIF_{+OA+CH}$ media)

TIM-1 protocol

Description of method

A standard fed state protocol was used. The half-life of gastric emptying was set at 80 min for both meals, and the gastric pH was programmed to drop from 5. 5 to 2.7 ± 0.2 during the first 60 min and from 2.7 to 1.5 ± 0.2 during the next 120 min. Gastric secretions in the model, which included digestive enzymes and hydrochloric acid, enabled the pH change in the gastric compartment. The intestinal (duodenal) pH under fasted and fed state was maintained at 6.5 ± 0.2 by the model secretions, which included sodium bicarbonate and digestive enzymes. The pH in the jejunum and ileum compartments of TIM-1 were similarly maintained at 6.8 ± 0.2 and 7.2 ± 0.2, respectively. Moderate and high fat meals to mimic those administered to humans were prepared and homogenised prior to the experiment. The dosage form was introduced into the model at the start of the experiment with the homogenised meal. The fed state experiments were run for a total of 5 hours with samples taken after filtration through a lipid ultrafiltration membrane from the jejunum and ileum filtrate at 0, 15, 30, 45, 60, 90, 120, 150, 180, 210, 240, 270 and 300 minutes. TIM-1 samples were analysed by HPLC on the same day the experiments were run.

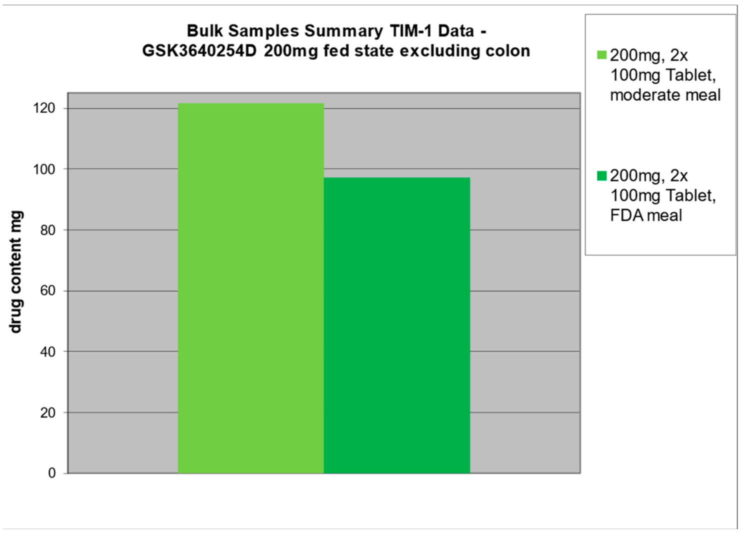

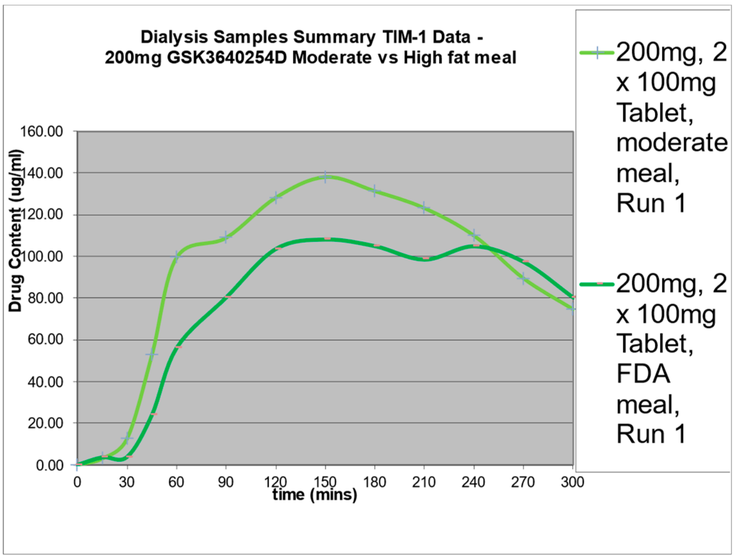

Figure S6. Comparison of moderate and high fat (FDA) meals (200mg:2x100mg tablets) on the bioaccessibility of GSK254 using TIM-1

References

1. Sugano, K., *Theoretical investigation of passive intestinal membrane permeability using Monte Carlo method to generate drug-like molecule population.* International Journal of Pharmaceutics, 2009. **373**(1): p. 55-61.
2. Joshi, S.R., et al., *Phase I evaluation of the safety, tolerability, and pharmacokinetics of GSK3640254, a next-generation HIV-1 maturation inhibitor.* Pharmacology research & perspectives, 2020. **8**(6): p. e00671-e00671.
3. Wen, B., et al., *Investigation of Clinical Absorption, Distribution, Metabolism, and Excretion and Pharmacokinetics of the HIV-1 Maturation Inhibitor GSK3640254 Using an Intravenous Microtracer Combined with EnteroTracker for Biliary Sampling.* Drug Metab Dispos, 2022. **50**(11): p. 1442-1453.
